# Long-term outcomes in heritable thoracic aortic disease

**DOI:** 10.3389/fcvm.2022.1009947

**Published:** 2022-10-13

**Authors:** Elizabeth N. Robertson, Paul G. Bannon, Richmond W. Jeremy

**Affiliations:** ^1^Central Clinical School, Faculty of Medicine and Health, University of Sydney, Sydney, NSW, Australia; ^2^Department of Cardiology, Royal Prince Alfred Hospital, Camperdown, NSW, Australia; ^3^The Baird Institute, Camperdown, NSW, Australia; ^4^Department of Cardiothoracic Surgery, Royal Prince Alfred Hospital, Camperdown, NSW, Australia

**Keywords:** Marfan, Loeys-Dietz, genetic, aneurysm, dissection, prognosis

## Abstract

Heritable aortic aneurysm is an increasingly recognized cause of morbidity and mortality. Whilst Marfan syndrome (MFS) is well-known, the clinical presentation and prognosis of more newly described genetic syndromes is less familiar to clinicians. There is a particular lack of knowledge regarding clinical outcomes for non-syndromal heritable aortic disease. This study investigated the presentation, clinical course and survival of patients with syndromal [Loeys-Dietz, aneurysm-osteoarthritis, and aneurysm-cerebral arteriopathy (*ACTA2*) syndrome] and non-syndromal heritable aortic disease in comparison to MFS. The study group includes 536 individuals (283 Marfan, 176 non-syndromal heritable aortopathy, 36 aneurysm-osteoarthritis, 32 Loeys-Dietz, and 9 *ACTA2* aneurysm) enrolled in a longitudinal clinical follow-up between 1990 and 2022. Age at diagnosis differed between groups: Marfan = 22.0 ± 16.6; Loeys-Dietz = 29.6 ± 21.5; aneurysm-osteoarthritis = 36.4 ± 18.8; *ACTA2* aneurysm = 43.4 ± 18.6; non-syndromal heritable aortopathy = 47.2 ± 16.6 years (*p* < 0.001). Aortic dissection was the presenting event in 8% individuals with Marfan compared to 27% with non-syndromal heritable aortopathy and 34% with Loeys-Dietz syndrome (*p* < 0.01). Mean follow-up duration for the group was 16.4 years (range 0.2–30 years) and 74 individuals died during follow-up (Marfan = 52, Loeys-Dietz = 6, aneurysm-osteoarthritis = 4, ACTA2 aneurysm = 1, heritable non-syndromal aortopathy = 11). At 10 years follow-up, actuarial mean survivals were: aneurysm-osteoarthritis = 77.5 ± 10.4%; Loeys-Dietz = 90.0 ± 6.8%; Marfan = 94.6 ± 1.4%; heritable non-syndromal aortopathy = 95.9 ± 2.1% (NS). There were 60 aortic dissections (24 Type A, 36 Type B) during follow-up. At 10 years, survival free of dissection was comparable between groups: aneurysm-osteoarthritis = 90.7 ± 6.4%; Loeys-Dietz = 94.4 ± 5.4%; Marfan = 96.1 ± 1.2%; heritable non-syndromal aortopathy = 93.9 ± 2.3%, with similar findings at 20 years. Prophylactic aortic surgery was a first event during follow-up for 196 individuals (ACTA2 aneurysm = 3; aneurysm-osteoarthritis = 10; Loeys-Dietz = 19; Marfan = 119; heritable non-syndromal aortopathy = 45). A second surgical intervention was required in 45 individuals and a third intervention in 21 individuals. At 10 years follow-up, survival free of surgery differed between groups: aneurysm-osteoarthritis = 68.5 ± 10.1%; Loeys-Dietz = 40.8 ± 11.2%; Marfan = 75.5 ± 2.7%; heritable non-syndromal aortopathy = 63.8 ± 4.7% (*p* < 0.001). At 20 years follow-up mean survival free of surgery was: aneurysm-osteoarthritis = 26.6 ± 14.7%; Loeys-Dietz = 9.1 ± 8.2%; Marfan = 57.2 ± 3.4%; heritable non-syndromal aortopathy = 41.6 ± 8.2% (*p* < 0.001). Diagnosis of newer syndromic and non-syndromal heritable aortopathies is delayed compared to MFS, with associated complications of presentation with aortic dissection. Survival of individuals enrolled in follow-up surveillance is comparable between different genetic aortopathies, however aortic dissections still occur and need for surgical intervention is high.

## Introduction

Thoracic aortic aneurysm disease (TAAD) is a significant cause of cardiovascular morbidity and mortality (1, 2). Commonly regarded as a disease of the older population, associated with risk factors of hypertension and atherosclerosis, it is now increasingly recognized that TAAD can cause aortic dissection and premature death in younger people, including adolescents (3). The substrate for disease in the younger cohort is a heritable aortopathy, consequent upon pathogenic variants in genes encoding key structural and signaling proteins in the aortic vascular smooth muscle cells (4). An increasing number of genes have been implicated, although many are yet described in relatively small patient cohorts. The best characterized genes include *FBN1* [Marfan syndrome (MFS)], *TGFBR1* and *TGFBR2* [Loeys-Dietz syndrome (LDS)], *SMAD3* [aneurysm-osteoarthritis syndrome (AOS)], and *ACTA2* [Aortic and Cerebral Aneurysm (ACA)]. A common feature of heritable TAAD is an autosomal dominant pattern of inheritance.

Whilst MFS is well-known, the other entities are less familiar to many clinicians. Key questions about these more recently described conditions include natural history and risk of complications, prognosis and life expectancy after an aortic dissection or aortic surgery. The evidence base providing answers to these questions continues to evolve. There is a particular need for comparative long-term longitudinal data about clinical outcomes for individuals with heritable aortic disease.

At the same time, there are many patients who present with thoracic aneurysm or dissection at a young age, for whom no underlying pathogenic gene variant is identifiable. Despite this, these patients often have a strong family history of TAAD indicating the genetic trigger likely exists but is unknown. This cohort is characterized as non-syndromal heritable TAAD (H-TAAD), however their clinical course and prognosis remains poorly characterized.

This study examines the clinical course and outcomes for cohorts of patients with the more common forms of heritable TAAD and also the outcomes for the cohort with H-TAAD without a known pathogenic gene variant. The specific research questions addressed are (i) what is the clinical course of initial presentation with an aortic event; (ii) what are the clinical outcomes after initial aortic event and (iii) what are the differences in outcomes according to underlying gene variants?

## Materials and methods

### Patient groups

The data source was the clinical records of the Marfan and Aorta Clinic at Royal Prince Alfred Hospital (RPAH), Sydney, Australia. This clinic was established in 1988 for diagnosis and management on individuals with MFS and has since expanded to include individuals with all genetic aortic diseases. Patients attending the Clinic have a full medical and surgical history documented, physical examination and aortic imaging with echocardiography plus CT/MRI angiography of the aorta and additional vascular imaging according to clinical indication.

Individuals are referred to the Clinic because of clinical suspicion of a heritable aortopathy, or for diagnostic evaluation after an initial clinical event or for screening as an at-risk family member following diagnosis of a heritable aortopathy in a proband. Prospective data collection has been maintained since 1990 and includes documented genotype, phenotype, clinical events, operative records and aortic imaging. Data is recorded on a secure database, accessible only to the investigators. This study was a retrospective review of the data for all patients seen at the Clinic between January 1, 1990 and December 31, 2021. All individuals with a diagnosis of MFS, LDS, AOS, ACA, or H-TAAD are included in this study.

Genotyping was undertaken in consultation with the Clinical Genetics Service at RPAH or affiliated genetics services. After informed consent, testing of patient DNA for likely pathogenic variants was conducted using a panel of candidate genes, initially including *FBN1*, *FBN2*, *TGFBR1*, *TGFBR2*, *ACTA2*, *SMAD3*, *MYH11*, *MYLK*, and *COL3A1* and more recently extended to include *TGFB2*, *TGFB3*, *SMAD2*, *SMAD4*, *COL5A1*, *COL5A2*, *NOTCH1*, *FLNA*, *FBLN5*, *ELN*, *LOX*, and *SKI*. Identification of pathogenic variants was in accord with recommendations of the American College of Medical Genetics and Genomics (5).

Diagnosis of a heritable aortopathy was made according to the following criteria: MFS–revised Ghent criteria/pathogenic *FBN1* variant (6); LDS–consistent clinical features/pathogenic *TGFBR1* or *TGFBR2* variant (7); AOS–consistent clinical features/pathogenic *SMAD3* variant (8); aneurysm-cerebral arteriopathy syndrome (ACA)–aortic or cerebrovascular aneurysm/pathogenic *ACTA2* variant (9, 10). Individuals with TAAD, without predisposing factors or hypertension, and a confirmed family history of TAAD in a first relative at age <50 years, but no identifiable pathogenic gene variant were classified as H-TAAD-gene unknown. Exclusion criteria were: individuals diagnosed with one of the less common gene variants associated with a heritable aortopathy, e.g., *LOX*, *TGFB2* are not included due to small numbers described to date; individuals for whom bicuspid aortic valve (BAV) was the primary pathology, or individuals with isolated aortic aneurysm without a family history.

### Follow-up

Patient follow-up is recommended yearly for younger patients or those with recent diagnosis, and at intervals no greater than 2 years for older individuals with stable aortic geometry. Follow-up review includes clinical and echocardiographic examination, with additional CT or MR imaging as indicated. Follow-up was through the clinic and referring practitioners and is reported for follow-up through to the study end date of December 31, 2021. Fifteen individuals (all MFS) were lost to follow-up and for these individuals the survival data at last follow-up is reported. Major adverse cardiac events (MACE) during follow-up were aortic surgery, heart valve surgery, other cardiac surgery, ischemic stroke, cerebral hemorrhage, infective endocarditis and complications of anticoagulation.

Patients are recommended to have medical therapy with beta-adrenergic blockers and/or angiotensin receptor blockers according to clinical indications (11–13). Surgical intervention on aorta, aortic and mitral valves is recommended according to clinical circumstances and with reference to current clinical guidelines (14, 15). Specific indications for aortic surgery were an absolute aortic diameter (at aortic sinuses or ascending aorta) ≥50 mm (or≥45 mm for those with LDS), OR a rapidly increasing aortic diameter (>3 mm in 2 years) OR aortic diameter ≥43 mm in a woman planning pregnancy OR aortic diameter ≥43 mm in a patient with a strong family history of aortic dissection (2 or more first degree relatives with dissection). Management also includes advice on lifestyle and physical activity (16).

### Statistics

Continuous data are presented as mean ± standard deviation for normally distributed data and median with interquartile range for non-normal data. Data normality was tested by Kolmogorov–Smirnov test. Discrete data are presented as proportions or percentages. Comparisons of continuous data sets was by analysis of variance for normally distributed data and by Mann–Whitney *U* for non-normal data. Comparisons of discrete data were by Chi-square test or Fisher’s Exact Test (if cell *n* less than 5). Event-free survival for patient cohorts was described by Kaplan-Meier estimate with comparison between cohorts by log rank test. Kaplan-Meier analysis was not performed for the ACA group due to small numbers. Statistical analyses were performed using SPSS 27 (IBM, Armonk, NY, USA).

### Ethics

The use of aggregated, de-identified clinical data for outcomes research was conducted under the research protocol X15-0382, approved by the Human Research Ethics Committee of Sydney Local Health District. All patients gave written, informed consent prior to genotyping.

## Results

A total of 536 individuals are included in the data set, of whom 283 had MFS, 176 H-TAAD, 36 AOS, 32 LDS, and 9 ACA. The demographics of these groups are compared in Table 1. A marked sex imbalance was evident in H-TAAD, with 75% of individuals being male, compared to approximate male:female balance in other groups (*p* < 0.00001). Patients with MFS had a mean age of 22.1 years at diagnosis, but diagnosis was delayed in other groups, particularly those with H-TAAD, who had a mean age of 49.0 years at diagnosis (*p* < 0.00001). In all groups, the majority of affected individuals had a family history of TAAD (universal by definition in H-TAAD).

**TABLE 1:** Demographics of patient groups at presentation.

	Marfan	Loeys-Dietz	Aneurysm-osteoarthritis	Aneurysm-cerebral arteriopathy	H-TAAD gene unknown
Group *n*	283	32	36	9	176
M/F	165/118	16/16	18/18	4/5	133/43
Age diagnosis (years)	22.1 ± 16.6	30.7 ± 22.7	38.3 ± 18.1	45.6 ± 17.9	49.0 ± 16.7
Height (cm)	184 ± 12	177 ± 9	182 ± 12	171 ± 16	179 ± 10
Weight (kg)	75 ± 17	66 ± 14	86 ± 19	72 ± 15	87 ± 18
BSA (m^2^)	1.98 ± 0.24	1.81 ± 0.19	2.02 ± 0.27	1.86 ± 0.15	2.06 ± 0.22
Family history TAAD	192 (68%)	22 (69%)	30 (83%)	6 (66%)	176 (100%)
Smoker	11	0	2	0	3
Diabetes	2	1	0	0	1
Hypertension	1	0	2	0	2
**Presentation**					
Clinical suspicion	260	21	23	4	119
Aortic dissection	23	11	11	3	48
Cerebral hemorrhage	0	0	1	2	6
Sudden death	0	0	1	0	3

Mode of presentation also differed between groups. Among the MFS group, 23 (8%) presented with a major vascular event (aortic dissection, cerebral hemorrhage, or sudden death) compared to 5 (56%) ACA, 13 (36%) AOS, 11 (34%) LDS, and 57 (32%) H-TAAD (*p* < 0.00001). The observed mean ages of those presenting with a major vascular event were: ACA 41.8 ± 17.2 years; AOS 42.4 ± 18.8 years; LDS 39.9 ± 23.9 years; MFS 40.4 ± 11.8 years; H-TAAD 52.6 ± 14.8 years (*p* = 0.0003). Among the 57 H-TAAD patients presenting with a major vascular event 19 (33%) were aged >60 years. Among eight patients presenting with cerebral hemorrhage, five had H-TAAD.

**FIGURE 1:**
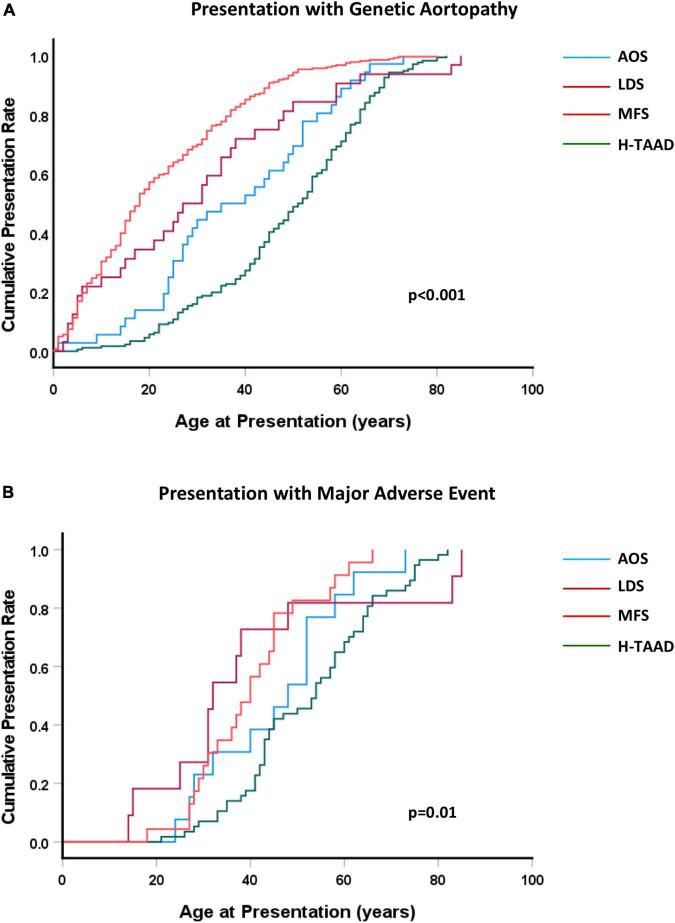
**(A)** Cumulative presentation rate, according to age, for different heritable aortopathies. **(B)** Cumulative presentation rate with MACE, according to age, for different heritable aortopathies. Abbreviations as per text.

The cumulative events rates for presentation are compared between groups in Figure 1A. Median ages at presentation vary from 22.2 ± 1.0 years for MFS to 48.9 ± 1.3 years (H-TAAD). At age 35 years, only 20% of H-TAAD individuals had been diagnosed, compared to 75% of those with MFS. Diagnosis after age 50 years was uncommon for MFS, yet half of those with H-TAAD were only diagnosed after age 50 years. Similarly, diagnosis for those with AOS was delayed to the 3rd decade or later for the majority of individuals. The cumulative event rates for presentation with a major adverse cardiovascular event are also shown in Figure 1B. Individuals with LDS presented at younger ages with a major adverse event, whilst those with MFS and H-TAAD presented 10–20 years later.

**FIGURE 2:**
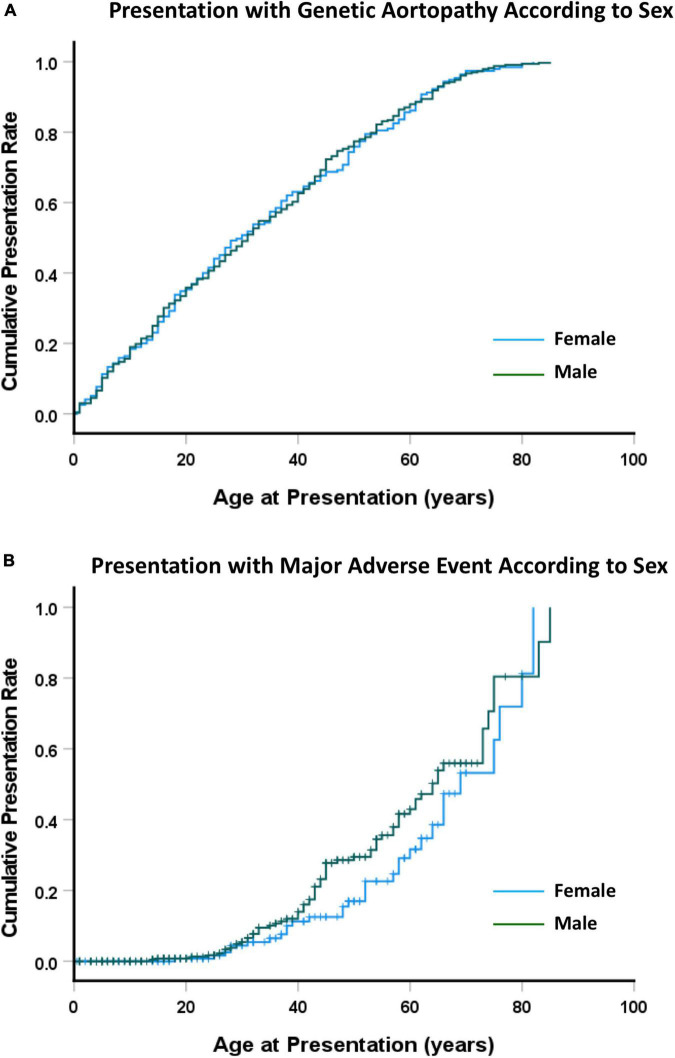
**(A)** Cumulative presentation rate, according to sex. **(B)** Cumulative presentation rate with MACE, according to sex. Abbreviations as per text.

Across the whole study group, there were no differences in presentation between males and females (Figure 2A), with median age at presentation of 30 ± 2.7 years for females and 31 ± 1.9 year for males (NS). There were 104 patients who presented with an adverse event (31 female, 73 male). The median age of presentation with an adverse event was 52 ± 4 years for females and 44 ± 1 years for males (NS) (Figure 2B). When presentation was examined by type of aortopathy, males with LDS or AOS had an earlier presentation than did those with MFS or H-TAAD, however the differences were not significant.

There were 50 fatal presentations (ACA 2; AOS 10; LDS 4; MFS 4; H-TAAD 30), with 486 individuals entering follow-up (ACA 7; AOS 26, LDS 28, MFS 279, H-TAAD 146). Mean follow-up durations of individuals after presentation were MFS 20.4 ± 11.4 years; LDS 15.0 ± 11.3 years; AOS 9.5 ± 5.8 years; ACA 12.4 ± 8.2 years; H-TAAD 10.3 ± 8.0 years (*p* < 0.00001).

**FIGURE 3:**
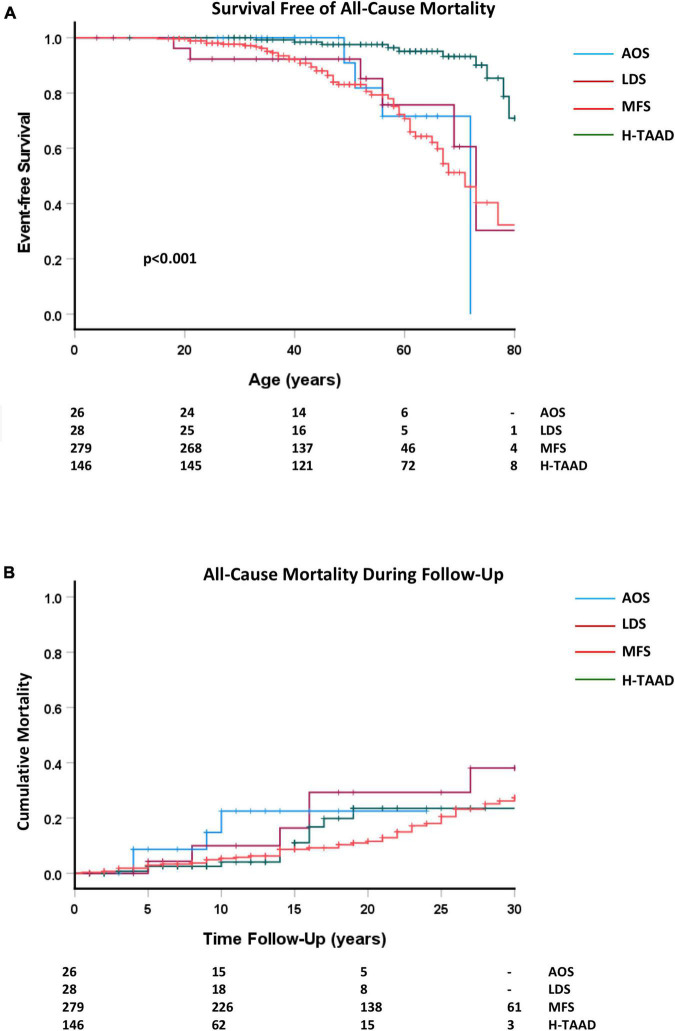
**(A)** Comparative event-free survival (all-cause mortality), according to age at death or last follow-up. **(B)** Comparative event-free survival (all cause-mortality), according to length of follow-up. Abbreviations as per text. Numbers at risk shown at bottom of plots.

Outcomes during follow-up are summarized for the groups in Table 2. There were 74 deaths during follow-up, with the majority (*n* = 52) occurring in those with MFS (*p* < 0.05). Survival (all-cause mortality) is compared between groups, according to patient age and length of follow-up, in Figure 3. Individuals with H-TAAD had greater mean age at death [81.8 ± 1.4 years than did those with AOS (66.4 ± 3.2 years), LDS (68.5 ± 4.5 years) or MFS 68.6 ± 2.2 years]. At 10 years follow-up, mean actuarial survivals were: AOS = 77.5 ± 10.4%; LDS = 90.0 ± 6.8%; MFS = 94.6 ± 1.4%; H-TAAD = 95.9 ± 2.1% (NS). At 20 years follow-up, mean survivals were: AOS = 77.5 ± 10.4%; LDS = 70.7 ± 11.2%; MFS = 88.4 ± 2.2%; H-TAAD = 76.5 ± 6.9%.

**TABLE 2:** Clinical outcomes during follow-up.

	Marfan	Loeys-Dietz	Aneurysm-osteoarthritis	Aneurysm-cerebral arteriopathy	H-TAAD gene unknown
Group *n*	279	28	26	7	146
M/F	163/116	13/15	13/13	2/5	109/37
Age start follow-up	22.0 ± 16.6	29.6 ± 21.5	36.4 ± 18.8	43.4 ± 18.6	47.2 ± 16.6
Duration follow-up	20.4 ± 11.4	15.0 ± 11.3	9.5 ± 5.8	12.4 ± 8.2	10.3 ± 8.0
**Arterial dissection**					
Type A aorta	15	1	1	2	5
Type B aorta	29	2	1	2	2
Abdominal aorta	1	0	0	0	1
Carotid/vertebral	0	1	0	0	0
Ilio-femoral	2	0	0	0	0
**Aortic surgery**					
Ascending aorta	114	17	9	4	43
Arch	17	7	3	1	17
Descending aorta	19	5	1	1	6
Thoracoabdominal	10	3	0	0	1
Mitral surgery	27	3	1	1	2
Endocarditis	8	0	0	0	0
Stroke	5	0	0	1	6
Death	52 (19%)	6 (21%)	4 (15%)	1 (14%)	11 (8%)

A total of 333 non-fatal MACE occurred during follow-up (ACA 8, AOS 11, LDS 28, MFS 219, H-TAAD 67). Multiple adverse events were common with two adverse events in 70 individuals (ACA 3, LDS 6, MFS 50, H-TAAD 11) and three adverse events in 37 individuals (ACA 1, LDS 3, MFS 32, H-TAAD 1). Multiple adverse events were more likely in MFS than in the other aortopathies. Adverse events included mitral valve surgery (*n* = 34, MFS = 27), endocarditis (*n* = 8, all MFS), stroke/cerebral hemorrhage (*n* = 12, MFS = 5, H-TAAD = 6). A first adverse event occurred in 250 individuals during follow-up (ACA = 5; AOS = 13; LDS = 21; MFS = 152; H-TAAD = 59) of which 24 events were fatal (ACA = 1; AOS = 2; LDS = 2; MFS = 15; H-TAAD = 4). The age of first adverse event was ACA = 54.4 ± 19.3 years; AOS = 44.0 ± 18.7 years; LDS = 35.5 ± 18.1 years; MFS = 35.4 ± 15.2 years; H-TAAD = 52.8 ± 15.8 years (*p* < 0.001).

**FIGURE 4:**
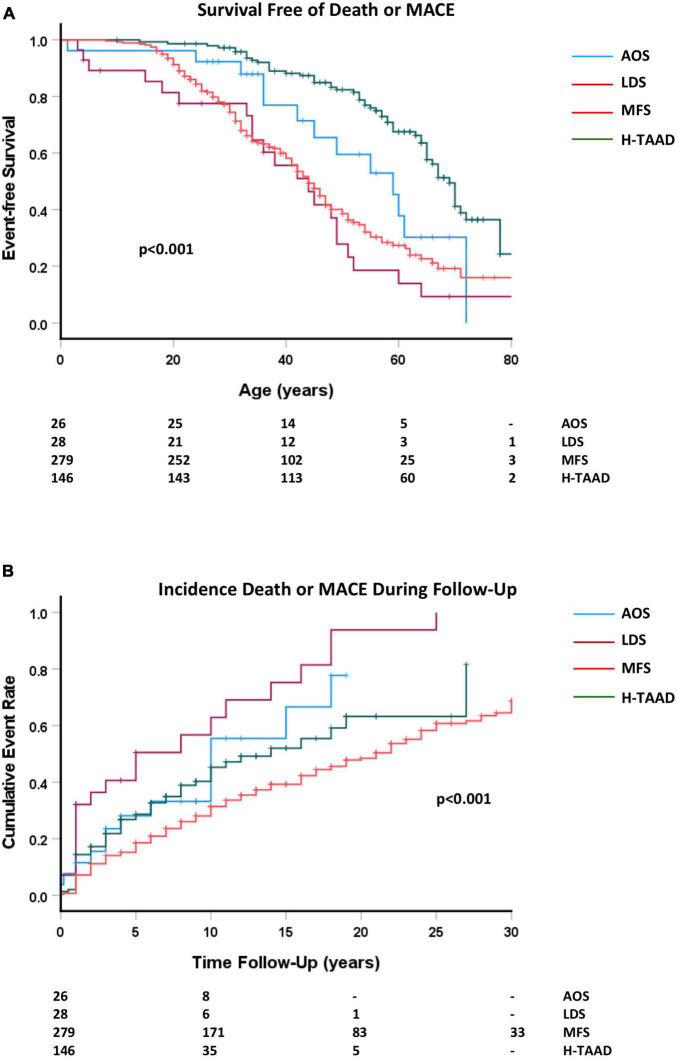
**(A)** Comparative survival free of death or major adverse cardiovascular event, according to age at event or last follow-up. **(B)** Cumulative event rate, for death or MACE, according to length of follow-up. Abbreviations as per text. Numbers at risk shown at bottom of plots.

Overall survival free of death or MACE is compared between groups in Figure 4. Individuals with AOS and H-TAAD had longer event-free survival than did those with LDS or MFS. Actuarial median survivals free of death or MACE were: AOS = 59 ± 6 years; LDS = 44 ± 5 years; MFS = 44 ± 2 years, H-TAAD = 69 ± 2 years. At 10 years follow-up, mean survival free of death or MACE differed between groups: AOS = 66.8 ± 9.8%; LDS = 37.1 ± 10.8%; MFS = 71.9 ± 2.8%; H-TAAD = 59.7 ± 4.7% (*p* < 0.001). At 20 years follow-up, mean survivals were: LDS = 6.2 ± 5.9%; MFS = 51.5 ± 3.4%; H-TAAD = 36.8 ± 7.3% (*p* < 0.001).

**FIGURE 5:**
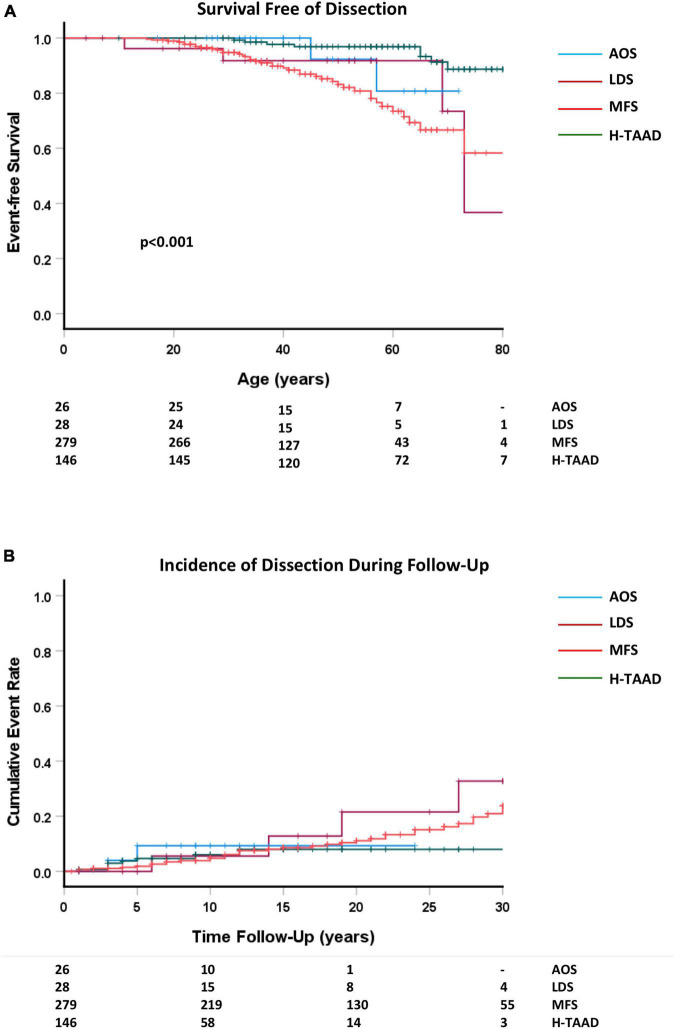
**(A)** Comparative survival free of dissection, according to age at dissection or last follow-up. **(B)** Cumulative event rate for any dissection, according to length of follow-up. Abbreviations as per text. Numbers at risk shown at bottom of plots.

There were 60 aortic dissection events (24 Type A, 36 Type B) and 5 other arterial dissections during follow-up, with the majority (*n* = 47) occurring in those with MFS (*p* < 0.001). Aortic dissection occurred as a first adverse event in 32 individuals (ACA = 2; AOS = 1; MFS = 20; H-TAAD = 9). Of those with an initial presentation with dissection, recurrent dissection during follow-up occurred in four patients (AOS = 1; MFS = 2; H-TAAD = 1). There were 29 Type B dissections during follow-up in the MFS group, of which 19 occurred after previous aortic root and ascending aorta repair (mean interval 9 years, range 2–18 years). Survival free of dissection, for individuals in follow-up, is compared between groups in Figure 5. At age 60 years, mean freedom from dissection was: AOS = 80.8 ± 12.6%; LDS = 91.8 ± 5.6%; MFS = 73.5 ± 4.4%; 96.9 ± 1.5% (*p* < 0.001).

**FIGURE 6:**
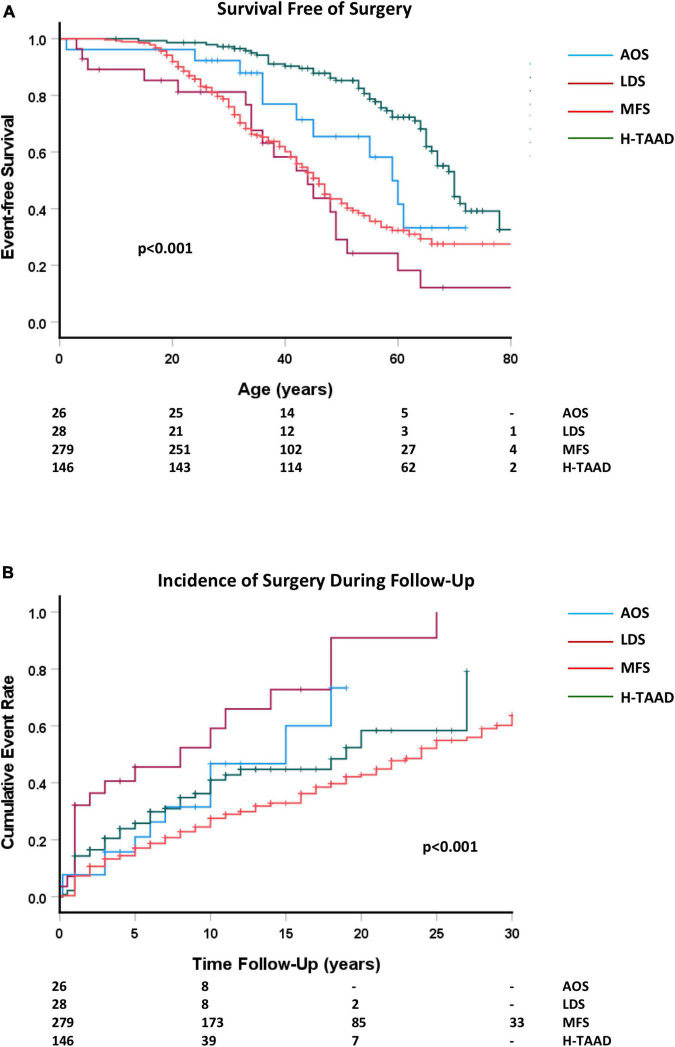
**(A)** Comparative survival free of any surgery, according to age at surgery or last follow-up. **(B)** Cumulative event rate for any surgery, according to length of follow-up. Abbreviations as per text. Numbers at risk shown at bottom of plots.

All patients undergoing cardiac or aortic surgery were included in subsequent follow-up. Survival free of any surgery as a first event is compared between aortopathy groups in Figure 6. Surgical procedures during follow-up included aortic root, ascending aorta, arch and descending aorta replacement; aortic or mitral valve replacement and repair of congenital defects (ventricular septal defect, patent ductus arteriosus).

There were no operative deaths or strokes related to elective repair of the aorta in absence of dissection, nor to aortic or mitral valve surgery. Two patients (LDS = 1; MFS = 1) suffered paraplegia after elective thoracoabdominal repair of previous descending aorta dissection. Two MFS patients died during emergent surgery for Type A dissection. Two patients (MFS = 1; H-TAAD = 1) died following elective thoracoabdominal repair of previous descending aorta dissection. Individuals with LDS and MFS required surgery more often and at younger age than did those with AOS or H-TAAD. Actuarial median survival free of surgery was: AOS = 59 ± 4 years; LDS = 44 ± 5 years; MFS = 46 ± 2 years; H-TAAD = 70 ± 2 years (*p* < 0.001). At 10 years follow-up, survival free of surgery differed between groups: AOS = 68.5 ± 10.1%; LDS = 40.8 ± 11.2%; MFS = 75.5 ± 2.7%; H-TAAD = 63.8 ± 4.7% (*p* < 0.001). At 20 years follow-up mean survival free of surgery was: AOS = 53.3 ± 12.3%; LDS = 9.1 ± 8.2%; MFS = 57.2 ± 3.4%; H-TAAD = 41.6 ± 8.2% (*p* < 0.001).

**FIGURE 7:**
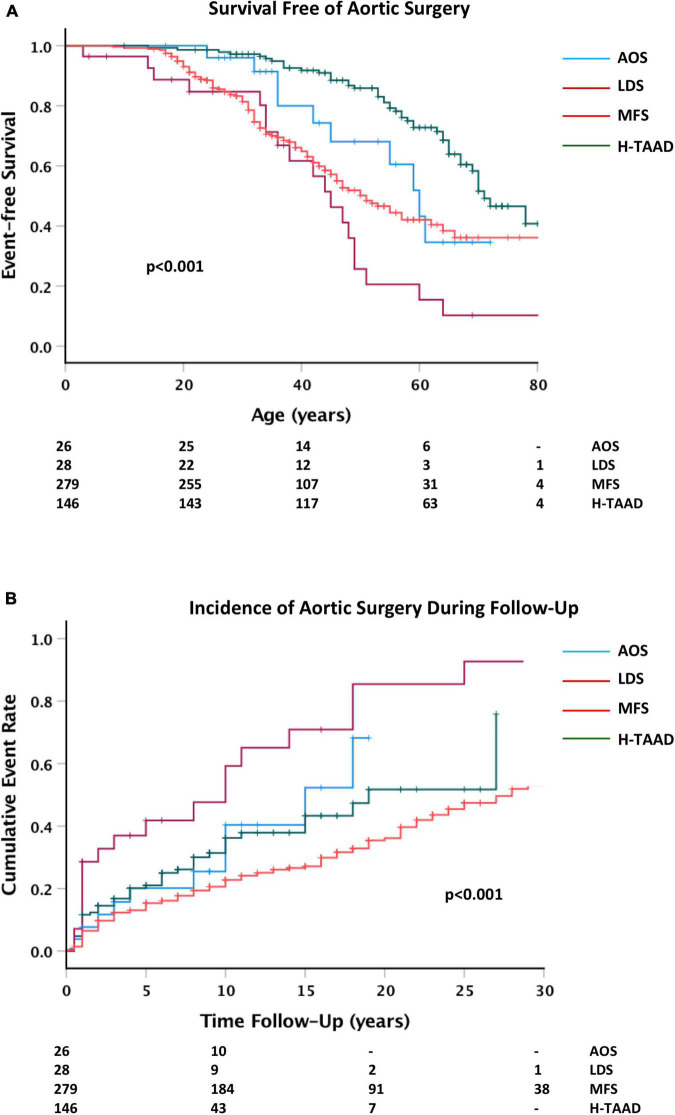
**(A)** Comparative survival free of aortic surgery, according to age at surgery or last follow-up. **(B)** Cumulative event rate for aortic surgery, according to length of follow-up. Abbreviations as per text. Numbers at risk shown at bottom of plots.

Elective prophylactic aortic surgery (i.e., not related to repair of acute dissection) was a first event during follow-up for 196 individuals (ACA = 3; AOS = 10; LDS = 19; MFS = 119; H-TAAD = 45). There were 45 individuals who required a second surgical intervention, not associated with dissection during follow-up (ACA = 1; LDS = 7; MFS = 27; H-TAAD = 10). A third operation was required in 21 individuals (MFS = 19; LDS = 2). Survival free of aortic surgery as a first event is compared between aortopathy groups in Figure 7. Actuarial median survival free of aortic surgery was: AOS = 59 ± 4 years; LDS = 44 ± 4 years; MFS = 57 ± 2 years; H-TAAD = 70 ± 2 years (*p* < 0.001). At 10 years follow-up, survival free of surgery differed between groups: AOS = 68.5 ± 10.1%; LDS = 52.4 ± 10.4%; MFS = 79.4 ± 2.4%; H-TAAD = 68.6 ± 4.5% (*p* < 0.001). At 20 years follow-up mean survival free of surgery was: AOS = 53.3 ± 12.3%; LDS = 14.5 ± 8.9%; MFS = 63.9 ± 3.3%; H-TAAD = 48.2 ± 7.5% (*p* < 0.001).

Outcomes according to medical treatment are summarized in Table 3 (ACA not included because of small numbers). Approximately half of patients in each group did not take any regular beta-blockers or angiotensin receptor blockers during follow-up, reflecting patient preference or intolerance to medication, particularly with hypotension in MFS. Both beta-blockers and angiotensin receptor blockers were taken by some patients (AOS = 2; LDS = 4; MFS = 57; H-TAAD = 31). Both age at entry to follow-up and duration of follow-up were comparable between those taking beta-blockers or angiotensin receptor blockers and those taking no medication in each aortopathy group. The incidence of aortic dissection during follow-up did not significantly differ between beta-blocker, angiotensin receptor blocker and nil treatment groups in any of the aortopathies. Similarly, treatment with either beta-blockers or angiotensin receptor blockers did not appear to change the need for surgical intervention, notably replacement of the ascending aorta. The incidence of death during follow-up was less for MFS patients taking either beta-blockers or angiotensin receptor blockers and for H-TAAD patients taking beta-blockers.

**TABLE 3:** Medications and clinical outcomes during follow-up.

	Marfan	Loeys-Dietz	Aneurysm-osteoarthritis	H-TAAD gene unknown
Nil treatment	127 (46%)	13 (46%)	16 (62%)	68 (47%)
M/F	60/67	5/8	7/9	48/20
Age start follow-up	22.9 ± 16.6	29.1 ± 21.2	40.7 ± 19.3	47.8 ± 17.1
Duration follow-up	19.1 ± 11.7	15.3 ± 11.1	7.3 ± 4.1	10.4 ± 7.2
Aortic dissection	20 (16%)	2 (15%)	1 (6%)	4 (6%)
Type A	9	1	1	3
Type B	11	1	0	1
**Aortic surgery**				
Ascending aorta	38 (30%)	9 (69%)	5 (31%)	20 (29%)
Arch	5	2	1	6
Descending aorta	4	2	0	3
Thoracoabdominal	2	2	0	1
Death	31 (24%)	4 (31%)	3 (19%)	8 (40%)
Beta blockers	120 (43%)	9 (32%)	3 (12%)	55 (38%)
M/F	80/40	6/3	2/1	24/11
Age start follow-up	19.6 ± 14.8	36.4 ± 26.8	38.7 ± 25.7	45.1 ± 17.2
Duration follow-up	21.3 ± 10.4	13.8 ± 11.4	10.7 ± 7.6	11.0 ± 9.6
Aortic dissection	22 (18%)	1 (11%)	0	3 (5%)
Type A	5	0	0	0
Type B	17	1	0	3
**Aortic surgery**				
Ascending aorta	58 (48%)	6 (67%)	0	18 (33%)
Arch	9	4	0	9
Descending aorta	11	2	1	2
Thoracoabdominal	8	1	0	0
Death	13 (11%) ^b^	2 (22%)	1 (33%)	1 (2%)^a^
Angiotensin blockers	88 (32%)	10 (36%)	9 (35%)	54 (37%)
M/F	62/26	5/5	6/3	44/10
Age start follow-up	22.4 ± 17.6	26.7 ± 15.1	27.0 ± 15.4	48.1 ± 16.1
Duration follow-up	21.3 ± 11.4	15.6 ± 12.0	13.9 ± 6.2	10.7 ± 9.1
Aortic dissection	13 (15%)	1 (10%)	0	4 (7%)
Type A	3	0	0	1
Type B	10	1	0	3
**Aortic surgery**				
Ascending aorta	38 (43%)	6 (60%)	4 (44%)	15 (28%)
Arch	7	4	2	9
Descending aorta	8	2	0	2
Thoracoabdominal	6	1	0	0
Death	11 (13%)^b^	1 (10%)	0	2 (4%)

^a^*p* < 0.05, ^b^*p* < 0.005 vs. nil treatment.

The outcomes in MFS and H-TAAD groups according to medical treatment are further described in Supplementary Table 1, with comparison between those taking nil treatment, beta-blockers only, angiotensin receptor blockers only or combined treatment. In MFS, the incidence of death during follow-up was lower in those taking both beta-blockers and angiotensin receptor blockers than in other treatment groups.

## Discussion

This study provides new information about clinical outcomes in patients with heritable aortic disease, particularly those with non-syndromic H-TAAD. Of particular note is the delayed diagnosis of individuals with H-TAAD and also the different clinical courses after diagnosis.

Our earlier study compared the clinical course of patients with BAV and MFS (17). Individuals with BAV had a low risk of death from aortic dissection, although a high rate of surgical intervention upon the aortic valve, consistent with other large scale longitudinal studies of BAV (18, 19). Given this established evidence base, BAV was not included in the present study. Our previous study did not include other syndromic aortopathies (ACA, AOS, and LDS) however increased clinical experience supports their inclusion in the present study. Although our earlier study included individuals with a clinical diagnosis on non-syndromal TAAD, the present study employs more stringent diagnostic criteria for H-TAAD with an absolute requirement for aortic disease in a first degree relative. We are now able to report longer follow-up times and more information about different clinical events during follow-up.

### Detection of heritable aortic disease

Individuals with un-diagnosed genetic aortopathy are at risk of undetected aneurysm progression and aortic dissection, with consequent morbidity and mortality. It is therefore of concern that we find delay in diagnosis for many individuals, particularly those with non-syndromal H-TAAD. Given the external physical features of MFS and LDS, it might be expected that the majority of affected individuals would be diagnosed at relatively young age. Notwithstanding this, 1 in 6 MFS and 1 in 4 LDS individuals were only diagnosed after age 40 years. For those with AOS and H-TAAD, half of those affected were not diagnosed until after age 40 years, yet the majority had a family history of TAAD. The issues with diagnosis of H-TAAD are further highlighted by the finding that individuals with H-TAAD are much more likely to present with aortic dissection (often fatal) than are those with other genetic aortopathies.

The increased risk of thoracic aortic disease in males is well-known, and previous studies have documented the differences between males and females in clinical expression of MFS (20, 21). Comparable data for the more recently described genetic aortopathies is less well established, however initial large studies show that males with *TGFBR1* variants present with aortic events at an earlier age than do females (7). In contrast, there does not appear to be a sex difference for individuals with *TGFBR2* or *ACTA2* variants.

The present study further uncovers the issue of sex for those with H-TAAD. Males are three times more likely to exhibit a phenotype of H-TAAD, despite observations of inheritance in an autosomal dominant manner. The reasons for this difference remain unknown. Experimental studies suggest that male sex hormones and an XY genotype contribute independently to increased risk of TAAD in males (22). The landscape is however more complicated by the occurrence of TAAD in females with Turner syndrome (23), which supports a gene dosage effect for genes on the X chromosome that escape inactivation. We have previously looked at potential candidates for such a gene dose effect, including *ACE2*, *FLNA*, and *EMD*. Whilst gene variants on the X chromosome are unlikely to be the primary cause of H-TAAD, they may have a modifying effect on phenotype. The difficulty in identifying causative variants in patients H-TAAD may in part be due to genotype positive, phenotype negative females, compounding association of phenotype to candidate gene variants.

The time course of presentation did not, however, differ between males and females and there was no difference between sexes in likelihood of presentation with a major adverse event. Our current data shows that females with phenotypic evidence of H-TAAD have a clinical course similar to that of males.

In the present study, 1 in 3 individuals with LDS or AOS presented with aortic dissection. In the large cohort with *TGFBR1/TGFBR2* variants described by Jondeau et al. (7), 1 in 6 affected individuals presented with aortic dissection. Of individuals with *SMAD3* variant associated AOS, who suffered aortic dissection, half had presented with dissection and did not have a genetic diagnosis prior to dissection (24). Among individuals with H-TAAD, 1 in 4 in the present series presented with aortic dissection.

Since initial description of LDS (25), the main clinical features in a large patient cohort have been described (7), however it is likely that many clinicians are still unfamiliar with the condition, particularly as it is less common than MFS. The same is likely true of more recently described aortopathies such as AOS (24, 26) and *ACTA2* related aneurysms and smooth muscle cell dysfunction (10). Some delay in diagnosis of these latter aortopathies is to be expected, given their relatively recent description. Nonetheless, the association of each condition with a family history of TAA stands out. Therefore, questions regarding an individual’s family history of TAA should be part of regular medical evaluations and are likely to improve detection of asymptomatic individuals and thereby save lives.

### Survival with heritable aortic disease

The survival of patients with MFS in the modern era has been described by our group and others (17, 19). Regular surveillance and targeted surgical intervention have improved life expectancy. More recently, cohort survival data for *TGFBR1/2* and *ACTA2* variant associated aortopathies have been described, however these previous studies have not provided in-depth information about clinical course after diagnosis, including risks of subsequent dissection and need for repeated surgical intervention (7, 10, 19). These are key questions for affected individuals and their medical attendants, for which the present study provides useful information.

Initial descriptions of LDS indicated that the condition was likely to be associated with markedly reduced life expectancy (25). The data provide by Jondeau et al. (7) in a larger cohort indicate that the prognosis is not as bleak as first appeared to be the case. Whilst those suffering a fatal aortic event appear most likely to do so by the fourth decade, the overall survival was >75% by age 70 years with no difference between *TGFBR1* and *TGFBR2* variant associated cases. Similarly, the present study shows survival at 60 years of age of 76 ± 12%. These findings are consistent with those of the GenTAC consortium (19). As with LDS, initial descriptions of AOS reported an aggressive cardiovascular phenotype (26). Our data shows that 60 years survival for those with AOS consequent upon *SMAD3* variants is similar to LDS at 72 ± 14%. Unfortunately, the large cohort study of Hostetler et al. (24) does not report overall survival. Interestingly, two-thirds of individuals with pathogenic *SMAD3* variants had no adverse aortic events. For those who had a fatal event, the median age was 56 years. Our data does not show any difference in overall survival between AOS, LDS and MFS. In contrast, overall survival in H-TAAD appeared significantly better at 95 ± 2% at age 60 and 93 ± 3% at age 70 years. This observation parallels that for outcomes in bicuspid valve aortopathy and interestingly similar observations have been made by the GenTAC consortium (19). When survival was compared between groups according to length of follow-up, the apparent advantage for H-TAAD was no longer evident. There are two possible explanations for this finding. In the first instance, the data may be skewed by a survivor effect, with other unrecognized patients with H-TAAD suffering fatal events prior to diagnosis. Alternately, the later presentation of H-TAAD, but similar course during follow-up may reflect a later age of onset of overt disease but similar pathological progression once disease is manifest. To date there is no comparable data for H-TAAD available in the literature and further investigation of this group will be warranted. It should be noted, however, that both the present study and other published studies have relatively small numbers of patients aged >60 years for LDS, AOS, and ACA. Specific further longitudinal studies of these groups is also required.

### Outcomes during surveillance

The present study provides novel insight into clinical outcomes for individuals enrolled in a dedicated surveillance and management program. Aortic dissection occurred in all groups during follow-up, with the majority observed in MFS. Notably, Type B dissection occurred as a late complication after aortic root and ascending aorta repair in patients with MFS. Surgical intervention (other than that required for management of an acute dissection) was required throughout follow-up in all groups and significant numbers of individuals required multiple interventions. There were differences between groups in the need for surgery. Those with LDS all required at least one surgery during follow-up, with half of these individuals requiring surgery within 5 years of presentation. In contrast, by 10 years follow-up over 75% of MFS and 65% of those with H-TAAD were free of need for surgery after presentation. Half of those with MFS had no need for surgery at even 25 years after initial presentation, however those MFS patients who had initial surgical requirements or dissection often had need for recurrent surgery. Outcomes for elective surgery in the absence of prior dissection are excellent, with no operative deaths or strokes occurring in this patient group.

### Implications for clinical practice

There are several findings from the present study, which impact upon clinical practice. The first is that a genetic aortopathy can present at any age and that the diagnosis of non-syndromic H-TAAD may often be missed. Therefore, clinicians should suspect an underlying genetic aortopathy for all patients presenting with thoracic aortic disease in the absence of a clear underlying cause such as severe hypertension or aortitis. A second key point, relevant to family screening, is that females with H-TAAD appear less likely to manifest aortic phenotypic features and they and their children may be erroneously classified as not being at risk. Thirdly, there is a significant risk of more distal aortic complications after initial aortic root and ascending aortic surgery, particularly for MFS. Finally, the excellent outcomes with elective aortic surgery support a strategy of earlier surgical intervention than current usual practice might recommend, particularly as aortic dissection is associated with significant ongoing morbidity and mortality after the initial event.

### Limitations and future directions

This study describes data prospectively collected over 30 years in a large aortic disease clinic. Many of the genetic aortopathies appear to be relatively uncommon and experience with less common gene variants is limited. Accordingly, multicentre datasets, such as those of the Montalcino Aortic Consortium, are going to be critical to further description of newly described genetic aortopathies and improved risk stratification. A limitation of both the present study and also of available evidence in the literature is small patient number after age 60 years, which underscores the need for further longitudinal studies with emphases on those in mid-life and older.

Improvements in genetic interrogation of individuals with TAAD, including better understanding of the role of non-coding regions of the genome, will expand the diagnostic repertoire and facilitate better classification of affected individuals. Improvements in genotype-phenotype correlations hold the promise of gene-directed management strategies and a further move toward personalized medicine.

An unresolved issue has been how much benefit accrues to adult patients from use of beta-adrenergic blockers and/or angiotensin-receptor blockers. Importantly, no study has found that either class of drugs confers harm to patients with genetic aortopathy, in contrast to calcium channel blockers (27). Conversely, there has been no evidence that either drug class is associated with a reduction in risk of death or adverse events. The present study did observe reduced incidence of death during follow-up in MFS patients taking either beta-blockers or angiotensin receptor blockers and H-TAAD patients taking beta-blockers. Whilst encouraging, these findings should be regarded with caution. This study was not a randomized trial and should not be regarded as definitive. There also remains debate about how much either drug class can reduce rates of aortic dilatation in patients with genetic aortopathy. It is our practice to inform patients of the available evidence and recommend use of beta-blockers in the first instance, provided patients can tolerate the medication. There is a clear need for further large-scale studies in different genetic aortopathies, as current evidence is based upon studies in those with MFS. Notably, patients with H-TAAD have stiffer aortas and higher blood pressure than those with MFS (28) and may derive greater benefit from beta-blocker treatment.

## Conclusion

A key message from the present study is the need for improved awareness of the genetic aortopathies among clinicians and heightened suspicion of involvement for any patient with a family history of thoracic aneurysm or dissection. Most importantly, clinicians should recognize that even an older patient presenting with thoracic disease may well have a genetic aortic disease, particularly of the non-syndromal form. Recurrent events after initial presentation or surgical repair of the aorta are frequent and all affected individuals should be enrolled in a life-long surveillance and management program. Nonetheless, survival for those enrolled in such a program is good and likely to improve further.

## Data availability statement

The original contributions presented in this study are included in the article/Supplementary material, further inquiries can be directed to RJ, richmond.jeremy@sydney.edu.au.

## Ethics statement

The studies involving human participants were reviewed and approved by Sydney Local Health District, Royal Prince Alfred Hospital. Written informed consent for participation was not required for this study in accordance with the national legislation and the institutional requirements.

## Author contributions

RJ: data collection and analysis, primary draft of manuscript, and final revision of manuscript. ER and PB: data collection, review and critical appraisal of manuscript, and approval of final submission. All authors contributed to the article and approved the submitted version.
