# Supplementary material for: Long-term outcomes in heritable thoracic aortic disease
Source: Front Cardiovasc Med. 2022 Oct 13;9:1009947. doi: 10.3389/fcvm.2022.1009947 (PMC9606219; doi:10.3389/fcvm.2022.1009947)
Supplement: Supplementary file 1 [file Table_1.pdf]

Supplementary Table 1 – Medications and Clinical Outcomes During Follow-Up in Marfan and H-TAAD Groups

	**Marfan**	**H-TAAD gene unknown**
**Nil Treatment**	127 (46%)	68 (47%)
**M / F**	60 / 67	48 / 20
**Age Start Follow-Up**	22.9 ± 16.6	47.8 ± 17.1
**Duration Follow-Up**	19.1 ± 11.7	10.4 ± 7.2
**Aortic Dissection**	20 (16%)	4 (6%)
**Type A**	9	3
**Type B**	11	1
**All Aortic Surgery**	49	30
**Ascending Aorta**	38 (30%)	20 (29%)
**Death**	31 (24%)	8 (40%)
**Beta Blockers Only**	63 (23%)	24 (14%)
**M / F**	40 / 23	17 / 7
**Age Start Follow-Up**	19.0± 14.3	43.7 ± 16.4
**Duration Follow-Up**	22.1 ± 10.6	9.4 ± 7.5
**Aortic Dissection**	13 (21%)	0
**Type A**	2	0
**Type B**	11	0
**All Aortic Surgery**	43	8
**Ascending Aorta**	31 (49%)	7 (29%)
**Death**	10 (16%)	1 (4%)
**Angiotensin Blockers Only**	31 (11%)	23 (16%)

**M / F**	22 / 9	17 / 6
**Age Start Follow-Up**	26.4 ± 20.8	50.7 ± 13.3
**Duration Follow-Up**	23.0 ± 13.3	8.7 ± 5.4
**Aortic Dissection**	4 (13%)	1 (4%)
**Type A**	0	1
**Type B**	4	0
**All Aortic Surgery**	16	5
**Ascending Aorta**	11 (35%)	4 (17%)
**Death**	8 (26%)	2 (8%)
**Combined Treatment**	57 (20%)	31 (21%)
**M / F**	40 / 17	17 / 4
**Age Start Follow-Up**	20.3 ± 15.3	46.2 ± 17.9
**Duration Follow-Up**	20.3 ± 10.2	12.2 ± 10.9
**Aortic Dissection**	9 (16%)	3 (10%)
**Type A**	3	0
**Type B**	6	3
**All Aortic Surgery**	43	21
**Ascending Aorta**	27 (47%)	11 (48%)
**Death**	3 (5%) [b]	0

[b] p<0.005 vs nil treatment.
